# Preparation and In Vitro Photodynamic Activity of Glucosylated Zinc(II) Phthalocyanines as Underlying Targeting Photosensitizers

**DOI:** 10.3390/molecules22050845

**Published:** 2017-05-19

**Authors:** Jian-Yong Liu, Chen Wang, Chun-Hui Zhu, Zhi-Hong Zhang, Jin-Ping Xue

**Affiliations:** 1 State Key Laboratory of Photocatalysis on Energy and Environment & National & Local Joint Biomedical Engineering Research Center on Photodynamic Technologies, College of Chemistry, Fuzhou University, Fuzhou 350108, China; n151320090@fzu.edu.cn (C.W.); zhuchunhui3333@sina.com (C.-H.Z.); xuejinping66@fzu.edu.cn (J.-P.X.); 2 Fuzhou General Hospital of Nanjing Military Command, Fuzhou 350005, China

**Keywords:** glucosylated phthalocyanine, photodynamic therapy, targeting photosensitizer, click chemistry

## Abstract

Two novel glucosylated zinc(II) phthalocyanines **7a**–**7b**, as well as the acetyl-protected counterparts **6a**–**6b**, have been synthesized by the Cu(I)-catalyzed 1,3-dipolar cycloaddition between the propargylated phthalocyanine and azide-substituted glucoses. All of these phthalocyanines were characterized with various spectroscopic methods and studied for their photo-physical, photo-chemical, and photo-biological properties. With glucose as the targeting unit, phthalocyanines **7a**–**7b** exhibit a specific affinity to MCF-7 breast cancer cells over human embryonic lung fibroblast (HELF) cells, showing higher cellular uptake. Upon illumination, both photosensitizers show high cytotoxicity with IC_50_ as low as 0.032 µM toward MCF-7 cells, which are attributed to their high cellular uptake and low aggregation tendency in the biological media, promoting the generation of intracellular reactive oxygen species (ROS). Confocal laser fluorescence microscopic studies have also revealed that they have high and selective affinities to the lysosomes, but not the mitochondria, of MCF-7 cells. The results show that these two glucosylated zinc(II) phthalocyanines are potential anticancer agents for targeting photodynamic therapy.

## 1. Introduction

Photodynamic therapy (PDT) is a unique non-invasive treatment for a range of cancers and non-cancerous diseases. It involves the combination of a photosensitizer (PS), light, and molecular oxygen (^3^O_2_). Upon irradiation, the excited PS transfers energy to the surrounding ^3^O_2_ to generate cytotoxic reactive oxygen species (ROS), especially singlet oxygen (^1^O_2_), which attacks the targeted cells, ultimately resulting in necrosis or apoptosis [1,2,3]. Phthalocyanines, as promising second-generation PSs for PDT, are of particular interest, owing to their suitable photo-physical and photo-chemical properties, such as the strong absorption in the red visible region (the Q band appears at ca. 670 nm with a molar absorptivity of ca. 10^5^ M^−1^·cm^−1^), red fluorescence emission approaching NIR wavelengths for deeper tissue imaging, high photo-stability and ^1^O_2_ quantum yield, and apparently low dark toxicity [4,5,6,7]. To date, a large number of phthalocyanine-based PSs have been prepared and evaluated for photodynamic activities, but most of them exhibit poor specific affinity to cancer cells. Thus, increasing the ability of PS to selectively target the malignant tumor cells, and especially the produce cytotoxicity without collateral damage, has been the most challenging study. The conjugation of PS with tumor specific vectors has been sought to cater to this strategy, including antibody, protein, peptide, transferrin, aptamer, folic acid, and biotin etc. [8,9,10,11,12,13,14,15,16,17,18,19,20]. 

Glucose transporters (GLUT) involved in the cellular uptake of carbohydrates are over-expressed in various carcinomas than in normal tissues [21], which are easily bound with glucose. It is considerable to combine the glucose as the target moiety with phthalocyanine to provide high therapeutic potency. Meanwhile, the amphiphilic capacity of PS as an important parameter for cellular uptake can also be optimized by binding hydrophilic glucose to the hydrophobic core of phthalocyanine. A variety of glycoconjugated phthalocyanines have been reported over the past decade [22,23]. They are usually obtained by deprotection of the protected glycoconjugated phthalocyanines. Unfortunately, some problems are often encountered by this procedure; for example, the deprotection is not completed or the deprotected products further undergo esterification with the acid in the reaction mixture. Therefore, the separation and purification has always been a bottleneck. Herein we describe the preparation of two novel glucoconjugated phthalocyanines by directly treating the propargylated phthalocyanine with azide-substituted glucoses via a Cu(I)-catalyzed “click reaction”. It is efficient to gain the satisfactory yield of the contrivable product and avoid the tedious separation and purification process by this method. Subsequently, we explored the photo-physical, photo-chemical, and photo-biological properties of these two compounds. The results show that the conjugates preferentially accumulate in the cancer cells and exhibit high photocytotoxicity with IC_50_ as low as 0.032 µM towards MCF-7 breast cancer cells. By these efforts, we enrich the family of asymmetric glucosylated phthalocyanines for PDT via a click reaction.

## 2. Results and Discussion

**Scheme 1:**
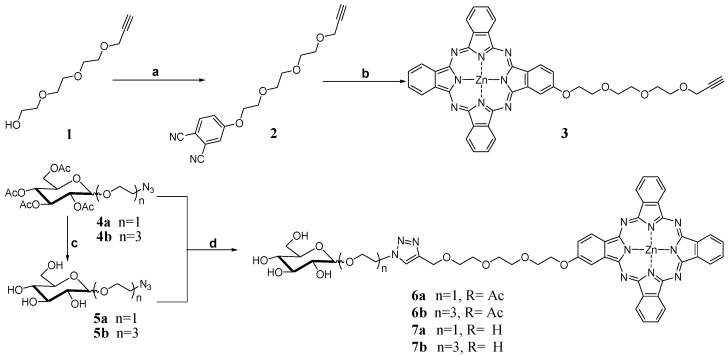
Synthesis of glucosylated zinc(II) phthalocyanines. *Reagents and conditions*: (**a**) 4-nitrophtalonitrile, K_2_CO_3_, DMF, 80 °C, overnight (65%); (**b**) phthalonitrile, Zn(OAc)_2_·2H_2_O, DBU, *n*-pentanol, 140–150 °C, overnight (22%); (**c**) CH_3_ONa, CH_3_OH, room temperature (r.t.), 2.5 h (95%); and (**d**) CuSO_4_·5H_2_O, sodium-ascorbate, CHCl_3_/C_2_H_5_OH/H_2_O (12:1:1, *v*/*v*/*v*), r.t., 24 h (56–63%).

The synthetic route used to prepare the glucosylated zinc(II) phthalocyanines **7a** and **7b** is depicted in Scheme 1. Firstly, treatment of triethyleneglycol with propargyl bromide and NaH resulted in monosubstitution giving compound **1** [24], which was then reacted with 4-nitrophthalonitrile in the presence of K_2_CO_3_ in *N*,*N*-dimethylformamide (DMF) to afford substituted phthalonitrile **2**. This compound then underwent statistical cyclotetramerization with unsubstituted phthalonitrile in the presence of Zn(OAc)_2_·2H_2_O and 1,8-diazabicyclo[5.4.0]undec-7-ene (DBU) in *n*-pentanol to give the propargylated phthalocyanine **3**.

Meanwhile, azide-substituted glucoses **5a** and **5b** were synthesized by treatment of the corresponding penta-acetate products **4a** and **4b** with sodium methoxide in CH_3_OH according to a procedure described in [25]. Finally, a Cu(I)-catalyzed “click reaction” between alkyne **3** and azides **5a**–**5b** was employed to prepare the glucosylated phthalocyanines **7a**–**7b**, in yields of 56–63%. For comparison, the acetyl-protected references **6a** and **6b** were also synthesized by treatment of the propargylated phthalocyanine **3** with the corresponding azide**s 4a** and **4b**, respectively.

### 2.1. Photo-Physical and Photo-Chemical Properties

**Figure 1:**
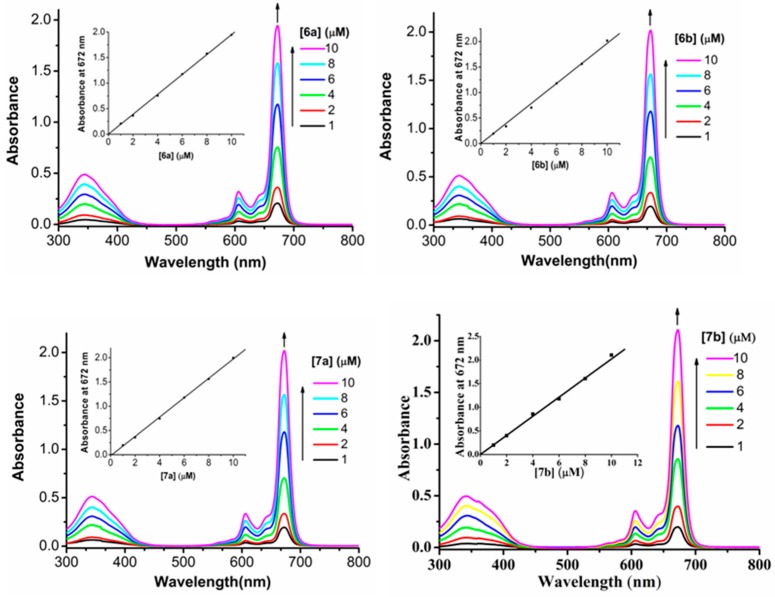
Electronic absorption spectra of **6a**–**6b** and **7a**–**7b** at various concentrations in DMF. The inset shows the variation of the Q-band absorbance with the concentration of phthalocyanine.

The electronic absorption, basic photo-physical and photo-chemical data of all these glucosylated zinc(II) phthalocyanines were measured in DMF and are summarized in Table 1. All of the compounds give typical absorption spectra of non-aggregated phthalocyanines, showing an intense and sharp Q-band at 672 nm, which strictly follows the Lambert-Beer’s law (Figure 1). Upon excitation at 610 nm, these compounds show a narrow fluorescence emission at 680 or 681 nm with a fluorescence quantum yield (Φ_F_) of 0.26–0.28 relating to unsubstituted zinc(II) phthalocyanine (ZnPc) (Φ_F_ = 0.28) (Table 1) [26]. To evaluate the photosensitizing potential, the singlet oxygen quantum yields (Φ_∆_) of these phthalocyanines were determined in DMF by a steady-state method using 1,3-diphenylisobenzofuran (DPBF) as the singlet oxygen scavenger and ZnPc as the standard [27]. It can be seen that all of these phthalocyanines are efficient singlet oxygen generators (Φ_∆_ = 0.63–0.65) (Table 1). These results show that effects of glucose moiety are negligible on the absorption, fluorescence emission, and singlet oxygen generation efficiency.

### 2.2. In Vitro Photo-Biological Properties

**Figure 2:**
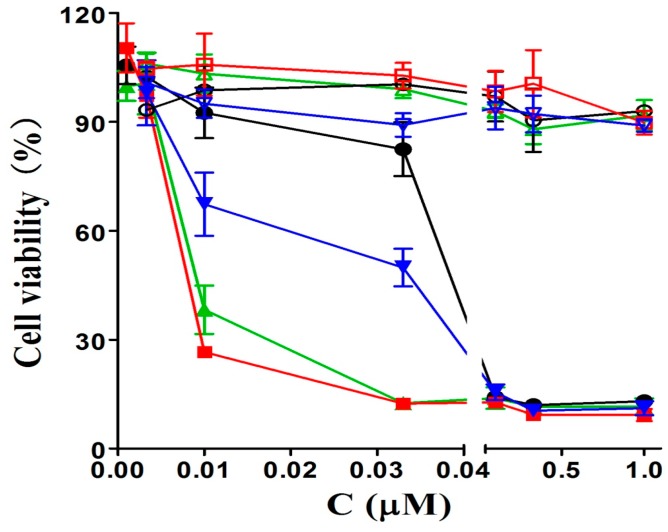
Effects of **6a** (■), **6b** (▲), **7a** (▼), and **7b** (●) on MCF-7 cancer cells in the absence (open symbols) and presence (closed symbols) of light (λ = 670 nm, 1.5 J/cm^2^). Data are expressed as mean values ± SD of three independent experiments, each performed in quadruplicate.

The in vitro photodynamic activities of glucosylated phthalocyanines **6a**–**6b** and the acetyl-protected **7a**–**7b** references toward human breast adenocarcinoma cell lines MCF-7, which overexpress GLUT, were determined by a modified methylthiazolyldiphenyltetrazolium bromide (MTT) assay. The corresponding dose-dependent survival curves are shown in Figure 2 and IC_50_ values, defined as the dye concentrations required to kill 50% of the cells, are summarized in Table 2. It is found that all these phthalocyanines have negligible cytotoxicity up to 1 µM in the absence of light. Upon irradiation of photosensitizer-stained MCF-7 cells with the light dose of 1.5 J/cm^2^ (λ = 670 nm), these compounds exhibit different degrees of photocytotoxicities. Phthalocyanines **7a** and **7b** having higher IC_50_ values (0.032 and 0.041 µM, respectively) show lower potency than **6a** and **6b** under the same experimental conditions.

**Figure 3:**
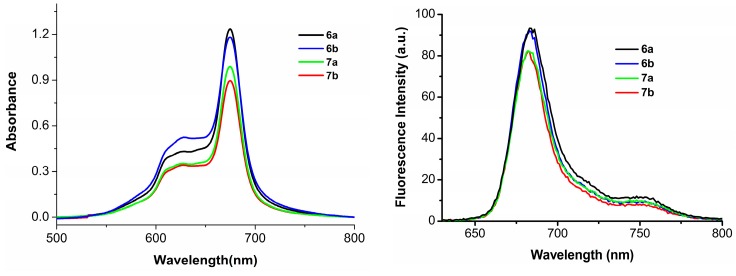
Electronic absorption (**left**) and fluorescence emission spectra (**right**) of **6a**–**6b** and **7a**–**7b**, formulated with Cremophor EL (0.04%) in the DMEM culture medium (all at 10 µM).

Having the large π-conjugated structure, phthalocyanine-based PSs always tend to aggregate in the aqueous solution, as a consequence, their photocytotoxicities are significantly reduced or even disappeared [28]. To account for the in vitro photodynamic activities, the aggregation behavior of phthalocyanines **6a**–**6b** and **7a**–**7b**, formulated with Cremophor EL in the Dulbecco’s modified Eagle’s medium (DMEM) culture medium, was examined by the electronic absorption and fluorescence spectroscopic methods. As shown in Figure 3, the Q-bands of all these compounds remain sharp and intense, meanwhile strong fluorescence emission peaks are observed at ca. 684 nm upon excitation at 610 nm indicating all these phthalocyanines are not significantly aggregated under these conditions, which seems to agree with their high photodynamic activities.

**Figure 4:**
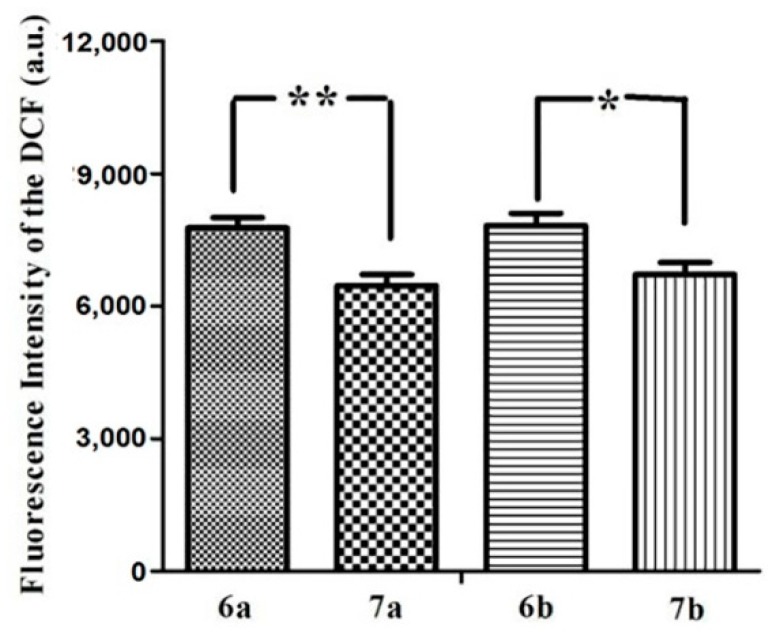
Cellular ROS generation efficiency for **6a**–**6b** and **7a**–**7b** (all at 5 µM) with the light dose of 1.5 J/cm^2^. Values are means ± SD. Statistical significance ** (*p* < 0.01), * (*p* < 0.05).

The irradiation of the photosensitizer leads to the production of ROS, which is thought to be the main mediator of cellular death induced by PDT, playing an important role in apoptosis [29]. It can mediate cellular effects such as lipid peroxidation and vascular effects, resulting in direct or indirect cytotoxic effects on the treated cells [30]. Here, the intracellular ROS generation efficiency of all of these glucosylated phthalocyanines against MCF-7 cells was also examined by using 2′,7′-dichlorodihydrofluorescein diacetate (DCFH-DA) as the indicator [31]. DCFH-DA is nonfluorescent, its oxidized product (DCF) by ROS can emit a green fluorescence. As shown in Figure 4, all of these phthalocyanines can sensitize the formation of ROS under illumination and the efficiency at generating ROS follows the order: **6a**–**6b** > **7a**–**7b**, which is in accordance with the in vitro photocytotoxicity.

**Figure 5:**
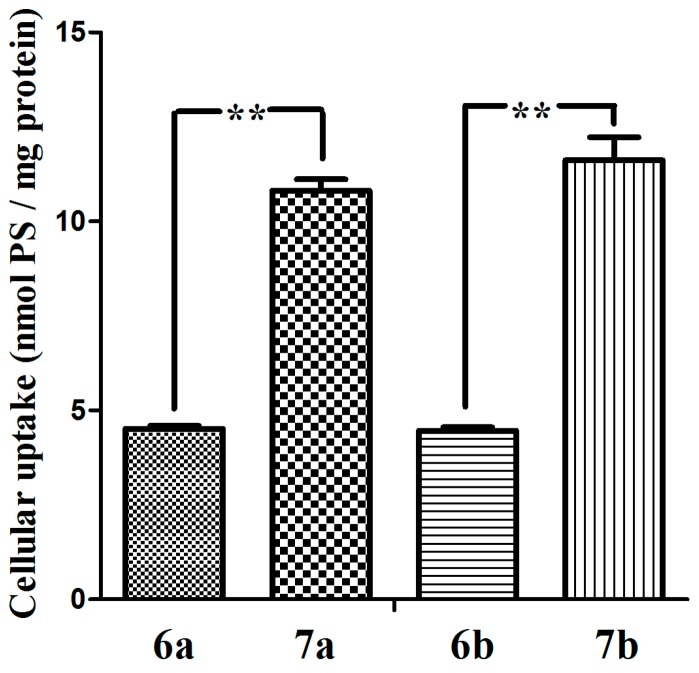
The cellular uptake of glucosylated zinc(II) phthalocyanines **7a**–**7b** and the references **6a**–**6b** (all at 5 µM) by MCF-7 cells (24 h). Statistical significance ** (*p* < 0.01).

We also investigated the cellular uptake of glucosylated phthalocyanines **7a**–**7b** and the acetyl-protected counterparts **6a**–**6b**. The intracellular amounts taken up by MCF-7 cells upon incubation with these dyes (5 µM, 37 °C, 24 h) were determined and expressed in nmol PS/mg cell protein. As shown in Figure 5 that the uptake of the glucosylated phthalocyanines **7a** and **7b** by MCF-7 cells are much more efficient than that of **6a** and **6b**. These results illustrate that the glucose of the conjugate enable it to be recognized specifically and taken up efficiently by the MCF-7 cancer cells. Interestingly, the conjugates **6a** and **6b** with the greater photodynamic activity correspond with the lower intracellular uptake, suggesting that uptake is not the only major factor involved in photodynamic damage. Moreover, differences in uptake between the dyes with different length of oligoethylene glycol chain are insensitive.

**Figure 6:**
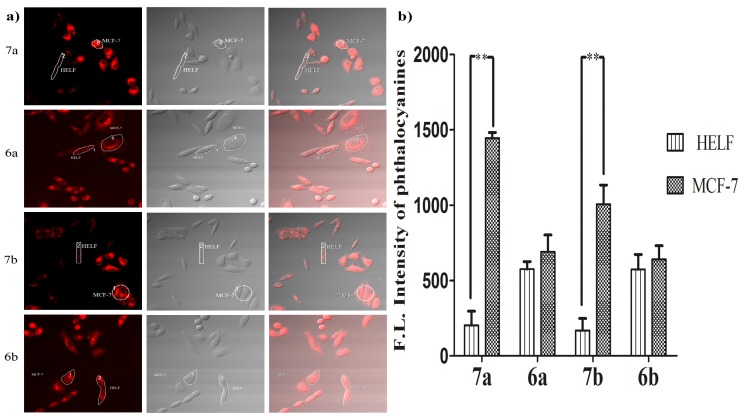
(**a**) Confocal fluorescence images of mixed MCF-7 and HELF cells after incubation with **7a**–**7b** and **6a**–**6b** for 24 h (all at 5 µM); (**b**) comparison of relative intracellular average fluorescence intensity of phthalocyanines in MCF-7 and HELF cells (measured in the ROIs). Data are expressed as means ± SD. Statistical significance ** (*p* < 0.01).

To further verify the specificity of the glucosylated zinc(II) phthalocyanines **7a** and **7b** to the cancer cells, a coculture experiment was designed. The strategy is to coculture cancerous MCF-7 and human embryonic lung fibroblast (HELF) cells which have significant differences in GLUT expression and morphology from each other in the same culture dish and to incubate these cells with glucosylated zinc(II) phthalocyanines **7a**–**7b** and the acetyl-protected references **6a**–**6b** (5 µM) for 24 h, respectively. The fluorescence caused by **7a**–**7b** and **6a**–**6b** (all excited at 633 nm, monitored at 650–750 nm) in these two cell lines was then recorded using a confocal laser scanning microscope. The confocal microscopy images clearly manifest that the fluorescence of **7a** and **7b** in MCF-7 cells is much higher than that in HELF cells (approximately seven-fold and five-fold, respectively) (Figure 6). However, the difference between MCF-7 and HELF cells for **6a** and **6b** is not obvious. The results show that **7a** and **7b** successfully targeted the cancer cells via high expression of GLUT.

**Figure 7:**
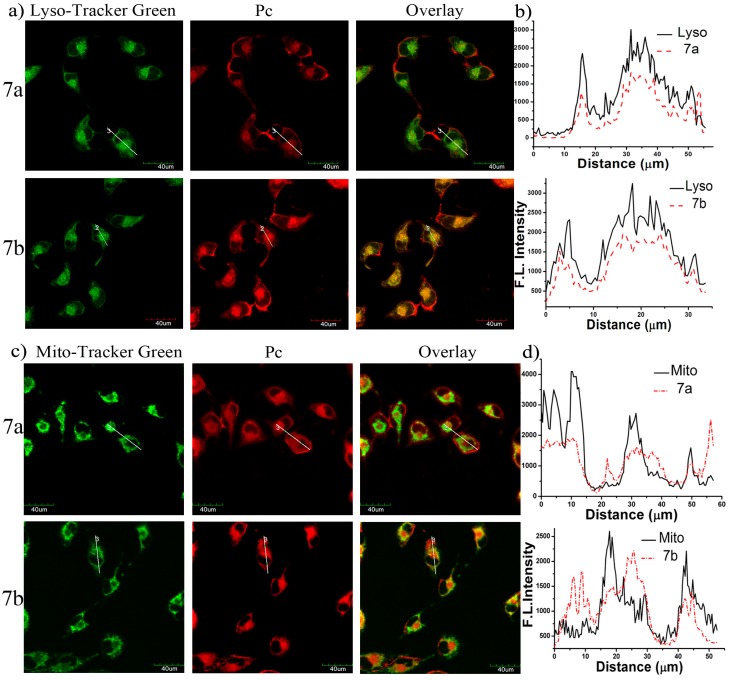
(**a**) Visualization of the intracellular fluorescence of MCF-7 cells for Lyso-Tracker (in green) and **7a** or **7b** (in red, both at 10 µM); (**b**) The fluorescence intensity profiles of Lyso-Tracker and **7a** or **7b** traced along the white line in (**a**). The corresponding images for **7a** or **7b** and Mito-Tracker Green are shown in (**c**,**d**).

The study of subcellular localization of **7a** and **7b** in MCF-7 cells was also carried out by an Olympus FV 1000 confocal laser scanning microscope (Olympus Instrument Co. Ltd., Shinjuku-ku, Tokyo, Japan). The cells were first incubated with the glucosylated zinc(II) phthalocyanines **7a**–**7b** in the culture medium for 24 h, and then stained with Lyso-Tracker Red DND 26 (for 30 min) or Mito-Tracker Green (for 30 min), which are specific dyes for lysosomes and mitochondria, respectively. It can be seen from Figure 7a that the fluorescence caused by the Lyso-Tracker (excited at 488 nm, monitored at 510–570 nm) can superimpose with the fluorescence caused by the photosensitizers **7a**–**7b** (excited at 633 nm, monitored at 650–750 nm). Furthermore, the very similar fluorescence intensity profiles (Figure 7b) traced along the white line also confirm that these two conjugates can target lysosomes of the cells. Conversely, the fluorescence images of **7a**–**7b** and the MitoTracker cannot be superimposed perfectly (Figure 7c,d), indicating that they are not well localized in the mitochondria but a bit distribution.

## 3. Experimental

### 3.1. General

All the reactions were performed under an atmosphere of nitrogen. Tetrahydrofuran (THF), methanol and *n*-pentanol were distilled from sodium. DMF was dried over barium oxide and distilled under reduced pressure. Dichloromethane was distilled from calcium hydride. Chromatographic purifications were performed on silica gel (Qingdao Ocean, Qingdao, Shandong, China, 200–300 mesh) columns with the indicated eluents. All other solvents and reagents were of analytical grade and used as received. Compounds **1** [24], **4a** and **4b** [32] were prepared as described.

^1^H-NMR spectra were recorded on a AVANCE III 400 (^1^H, 400; ^13^C, 100.6 MHz) instrument (Bruker, Karlsruhe, Germany) in CDCl_3_ or DMSO-*d*_6_; Chemical shifts were expressed in ppm relative to TMS (0 ppm). High-resolution mass spectra (HRMS) analyses were carried on a Agilent 6520 Accurate-Mass Q-TOF Mass Spectrometer (Agilent Technologies, Santa Clara, CA, USA). Electronic absorption spectra were measured on a Beijing PuXi Tu-1901 Spectrometer (Beijing PuXi Spectrum Optical Instrument Co. Ltd., Beijing, China) and fluorescence spectra were obtained on a VARIAN Carye Elipse Fluorescene Spectrometer (Agilent Technologies, Santa Clara, CA, USA). Subcellular localization was carried on Olympus FV1000 Confocal Laser Scanning Microscope (Olympus Instrument Co. Ltd., Shinjuku-ku, Tokyo, Japan). The purity of compounds **7a** and **7b** were found to be ≥95%. It was determined by analytical HPLC, which was performed on an waters 1525/2996 system equipped with an C-30 reverse phase column (4.6 mm × 250 mm, 5 µm) at a flow rate of 0.6 mL/min. Linear gradient of 20–10% acetonitrile (ACN) in dimethylacetamide (DMAC) over 15 min for compounds **7a** and **7b**.

### 3.2. Synthesis

#### 3.2.1. Propargylated Phthalonitrile (**2**)

A mixture of 4-nitrophthalonitrile (2.07 g, 12.0 mmol), compound **1** (1.88 g, 10.0 mmol), and K_2_CO_3_ (8.28 g, 60.0 mmol) in DMF (20 mL) was stirred at 80 °C for overnight. The volatiles were removed under reduced pressure. The residue was mixed with water (150 mL) and then extracted with CH_2_Cl_2_ (3 × 150 mL). The combined organic extracts were dried over anhydrous Na_2_SO_4_ and then concentrated to dryness. The residue was purified by silica gel column chromatography using CH_2_Cl_2_/CH_3_OH (100:1, *v*/*v*) as the eluent to give a pale yellow solid (2.05 g, 65%). ^1^H-NMR (400 MHz, CDCl_3_): δ 7.71 (d, *J* = 8.8 Hz, 1H, ArH), 7.32 (d, *J* = 2.4 Hz, 1H, ArH), 7.23 (dd, *J* = 8.8, 2.4 Hz, 1H, ArH), 4.23 (t, *J* = 4.4 Hz, 2H, CH_2_), 4.20 (d, *J* = 2.4 Hz, 2H, CH_2_-C≡C-), 3.89 (t, *J* = 4.4 Hz, 2H, CH_2_), 3.65–3.74 (m, 8H, CH_2_), 2.43 (t, *J* = 2.4 Hz, 1H, –C≡CH). ^13^C-NMR (100.6 MHz, CDCl_3_): δ 161.89, 135.06, 119.81, 119.46, 116.91, 115.61, 115.17, 106.87, 79.50, 74.55, 70.61, 70.31, 70.16, 68.96, 68.85, 68.56, 58.11. HRMS (ESI): *m*/*z* calcd. for C_17_H_18_N_2_NaO_4_ [M + Na]^+^: 337.1164, found 337.1152.

#### 3.2.2. Propargylated Phthalocyanine (**3**)

A mixture of compound **2** (0.22 g, 0.70 mmol), unsubstituted phthalonitrile (0.81 g, 6.3 mmol) and Zn(OAc)_2_·2H_2_O (0.77 g, 3.5 mmol) in *n*-pentanol (15 mL) was heated to 100 °C, then DBU (0.5 mL) was added. The mixture was stirred at 140–150 °C overnight. After a transient cooling, the volatiles were removed by rotary evaporation. The residue was dispersed in CHCl_3_ (100 mL), and the mixture was filtered by diatomaceous earth to remove part of the unsubstituted zinc(II) phthalocyanine. The filtrate was collected and concentrated to dryness in vacuo. The residue was purified by silica gel column chromatography using CHCl_3_/CH_3_OH (20:1, *v*/*v*) as the eluent. Then the crude product was further purified by size exclusion chromatography using THF as the eluent. The target product was obtained by recrystallization from a mixture of THF and petroleum ether as a blue-green solid (0.12 g, 22%). ^1^H-NMR (400 MHz, DMSO-*d*_6_): 9.07–9.13 (m, 3H, Pc-H_α_), 9.01 (d, *J* = 7.2 Hz, 1H, Pc-H_α_), 8.90–8.95 (m, 2H, Pc-H_α_), 8.62 (d, *J* = 8.4 Hz, 1H, Pc-H_α_), 8.01–8.17 (m, 7H, 1 Pc-H_α_, 6 Pc-H_β_), 7.44 (d, *J* = 8.4 Hz, 1H, Pc-H_β_), 4.58 (t, *J* = 4.4 Hz, 2H, CH_2_), 4.23 (d, *J* = 2.0 Hz, 2H, CH_2_-C≡C-), 4.11 (t, *J* = 4.4 Hz, 2H, CH_2_), 3.87 (t, *J* = 4.8 Hz, 2H, CH_2_), 3.75 (t, *J* = 4.8 Hz, 2H, CH_2_), 3.67–3.71 (m, 4H, CH_2_), 3.47 (t, *J* = 2.0 Hz, 1H, -C≡CH). HRMS (ESI): *m*/*z* calcd. for C_41_H_31_N_8_O_4_Zn [M + H]^+^: 763.1760, found 763.1727.

#### 3.2.3. Acetyl-Protected Counterpart (**6a**)

To a mixture of compound **3** (0.15 g, 0.20 mmol) and **4a** (0.10 g, 0.24 mmol) in CHCl_3_/EtOH/H_2_O (7 mL, *v*/*v*/*v* = 12:1:1) were added CuSO_4_·5H_2_O (10 mg, 0.040 mmol) and ascorbate sodium (16 mg, 0.081 mmol). The mixture was stirred at room temperature for 24 h. Then H_2_O (30 mL) was added to the reaction mixture and the solution was extracted with CHCl_3_ (3 × 30 mL). The combined organic extracts were washed by saturated brine, and dried over anhydrous Na_2_SO_4_. The crude product was purified by silica gel chromatography (CHCl_3_/MeOH = 5:1, *v*/*v*) to afford blue-green solid **6a** (0.13 g, 56%). ^1^H-NMR (400 MHz, DMSO-*d*_6_): δ 8.93–9.18 (m, 6H, Pc-H_α_), 8.71 (br s, 1H, Pc-H_α_), 8.26 (br s, 1H, Pc-H_α_), 8.02–8.16 (m, 6H, Pc-H_β_), 7.97 (s, 1H, triazole-H), 7.47 (d, *J* = 8.4 Hz, 1H, Pc-H_β_), 5.23 (t, *J* = 9.6 Hz, 1H, CH), 4.90 (t, *J* = 9.6 Hz, 1H, CH), 4.73–4.83 (m, 2H, CH_2_), 4.49–4.61 (m, 6H, CH_2_), 4.16 (dd, *J* = 4.8 Hz, 12.4 Hz, 1H, CH), 3.91–4.12 (m, 6H, 2CH_2_, 2CH), 3.86 (vt, *J* = 4.8 Hz, 2H, CH_2_), 3.74 (t, *J* = 4.8 Hz, 2H, CH_2_), 3.64–3.69 (m, 4H, CH_2_), 2.01 (s, 3H, CH_3_), 1.97 (s, 3H, CH_3_), 1.92 (s, 6H, CH_3_); HRMS (ESI): *m*/*z* calcd. for C_57_H_53_N_11_NaO_14_Zn [M + Na]^+^: 1202.2963, found 1202.2934.

#### 3.2.4. Acetyl-Protected Counterpart (**6b**)

According to the procedure described for **6a**, compound **3** (0.15 g, 0.20 mmol) was treated with **4b** (0.12 g, 0.24 mmol), CuSO_4_·5H_2_O (10 mg, 0.040 mmol) and ascorbate sodium (16 mg, 0.081 mmol) to give **6b** as a blue-green solid (0.16 g, 63%). ^1^H-NMR (400 MHz, DMSO-d_6_): 8.87–9.13 (m, 6H, Pc-H_α_), 8.68 (br s, 1H, Pc-H_α_), 7.98–8.23 (m, 8H, 1 triazole-H, 1 Pc-H_α_, 6 Pc-H_β_), 7.46 (br s, 1H, Pc-H_β_), 5.25 (t, *J* = 9.6 Hz, CH), 4.90 (t, *J* = 9.6 Hz, CH), 4.73–4.82 (m, 2H, CH_2_), 4.53–4.60 (m, 4H, CH_2_), 4.50 (t, *J* = 4.8 Hz, 2H, CH_2_), 4.17 (dd, *J* = 4.8 Hz, 12.4 Hz, 1H, CH), 4.06–4.12 (m, 2H, CH_2_), 3.98–4.04 (m, 1H, CH), 3.93–3.98 (m, 1H, CH), 3.85 (t, *J* = 4.8 Hz, 2H, CH_2_), 3.79 (t, *J* = 5.2 Hz, 2H, CH_2_), 3.74 (t, *J* = 5.2 Hz, 2H, CH_2_), 3.63–3.72 (m, 4H, CH_2_), 3.52–3.59 (m, 2H, CH_2_), 3.41–3.49 (m, 6H, CH_2_), 2.01 (s, 3H, CH_3_), 1.97 (s, 3H, CH_3_), 1.96 (s, 3H, CH_3_), 1.93 (s, 3H, CH_3_). HRMS (ESI): *m*/*z* calcd. for C_61_H_63_N_11_O_16_Zn [M + 2H]^2+^: 634.6867, found 634.6856; *m*/*z* calcd. for C_61_H_62_N_11_O_16_Zn [M + H]^+^: 1268.3667, found 1268.3629.

#### 3.2.5. Glucosylated Zinc(II)-phthalocyanine (**7a**)

According to the procedure described for **6a**, compound **3** (0.15 g, 0.20 mmol) was treated with the deprotected glucose **5a** (0.10 g, 0.40 mmol), CuSO_4_·5H_2_O (10 mg, 0.040 mmol) and ascorbate sodium (16 mg, 0.081 mmol) to give **7a** as a blue-green solid (0.11 g, 56%). ^1^H-NMR (400 MHz, DMSO-*d*_6_): δ 9.37–9.48 (m, 6H, Pc-H_α_), 9.27 (d, *J* = 8.0 Hz, 1H, Pc-H_α_), 8.91 (s, 1H, Pc-H_α_), 8.20–8.28 (m, 6H, Pc-H_β_), 8.18 (s, 1H, triazole-H), 7.79 (d, *J* = 8.0 Hz, 1H, Pc-H_β_), 5.13–5.18 (m, 1H, CH), 5.06–5.10 (m, 1H, CH), 5.02–5.06 (m, 1H, CH), 4.67–4.72 (m, 2H, CH_2_), 4.53–4.62 (m, 6H, CH_2_), 4.23–4.28 (m, 2H, CH_2_), 4.03–4.10 (m , 3H, CH, CH_2_), 3.86–3.93 (m, 1H, CH), 3.74–3.81 (m, 2H, CH_2_), 3.67 (t, *J* = 4.4 Hz, 2H, CH_2_), 3.61–3.65 (m, 4H, CH_2_), 3.53–3.58 (m, 2H, CH_2_), 3.02–3.18 (m, 3H, OH), 2.93–3.00 (m, 1H, OH); HRMS (ESI): *m*/*z* calcd. for C_49_H_46_N_11_O_10_Zn [M + H]^+^: 1012.2715, found 1012.2705.

#### 3.2.6. Glucosylated Zinc(II)-phthalocyanine (**7b**)

According to the procedure described for **6a**, compound **3** (0.15 g, 0.20 mmol) was treated with deprotected glucose **5b** (0.14 g, 0.42 mmol), CuSO_4_·5H_2_O (10 mg, 0.040 mmol) and ascorbate sodium (16 mg, 0.081 mmol) to give **7b** as a blue-green solid (0.13 g, 59%). ^1^H-NMR (400 MHz, DMSO-*d*_6_): 9.12–9.35 (m, 6H, Pc-H_α_), 8.96 (br s, 1H, Pc-H_α_), 8.55 (br s, 1H, Pc-H_β_), 8.09–8.26 (m, 6H, Pc-H_β_), 8.06 (s, 1H, triazole-H), 7.61 (br s, 1H, Pc-H_β_), 4.95–5.00 (m, 1H, CH), 4.86–4.94 (m, 2H, CH), 4.59–4.68 (m, 2H, CH_2_), 4.58 (br s, 2H, CH_2_), 4.45–4.54 (m, 3H, CH, CH_2_), 4.12–4.17 (m, 1H, CH), 4.03–4.09 (m, 2H, CH_2_), 3.76–3.85 (m, 4H, CH_2_), 3.68–3.73 (m, 2H, CH_2_), 3.61–3.67 (m, 4H, CH_2_), 3.47–3.58 (m, 10H, CH_2_), 3.01–3.17 (m, 3H, OH), 2.92–3.00 (m, 1H, OH); HRMS (ESI): *m*/*z* calcd. for C_53_H_54_N_11_O_12_Zn [M + H]^+^: 1100.3239, found 1100.3223.

### 3.3. Photo-Physical and Photo-Chemical Studies

Fluorescence quantum yield (Φ_F_) was determined by the following equation:

Φ_F(sample)_ = (*F*_sample_/*F*_ref_)(*A*_ref_/*A*_sample_)(*n*_sample_^2^/*n*_ref_^2^) Φ_F(ref)_ (1)

where *F*, *A*, and *n* are the integrated fluorescence area (λ_ex_ = 610 nm, area under the emission peak), the absorbance at the excitation wavelength (610 nm), and the refractive index of the solvent, respectively. The unsubstituted zinc(II) phthalocyanine (ZnPc) in DMF was used as the reference with a value of Φ_F_ = 0.28. The Φ_F_ measurements were performed using diluted solution (absorbance of photosensitizers at 610 nm are 0.03–0.05). Singlet oxygen quantum yield (Φ_Δ_) was measured using 1,3-diphenylisobenzofuran (DPBF) as the scavenger. A laser (670 nm, 20 mW) was used as the light source and ZnPc was selected as the reference (Φ_Δ_ = 0.56 in DMF).

### 3.4. In Vitro Photodynamic Activities

Cell viability was determined by the colorimetric MTT assay [33]. MCF-7 cells were seeded onto 96-well plates at 6000 cells per well and incubated overnight. Glucosylated phthalocyanines **7a**–**7b** and the references **6a**–**6b** were diluted to the needed concentration (0.001, 0.0033, 0.01, 0.033, 0.1, 0.33, 1 µM) and added to six plicate wells. After 24 h incubation, the old medium containing drugs was replaced by fresh medium and the cells were exposed to red light (λ = 670 nm, 1.5 J·cm^−2^). The cells after irradiation were incubated again for 24 h and then a MTT solution in PBS (10 µL, 4 mg·mL^−1^) was added to each well. The plates were incubated for another 4 h. The medium was removed carefully and DMSO (100 µL) was added to each well. The absorbance of the solution at 570 nm in each well was taken by a microplate reader. Six replicates were run, and experiments were repeated at least three times. For the dark toxicity, the procedures are almost the same as above, except that there is no irradiation. The survival curves were plotted as a function of concentration of dyes with GraphPad Prism 5.0 software (GraphPad Software Inc., La Jolla, CA, USA) and IC_50_ values were calculated.

### 3.5. Measurement of Intracellular ROS

ROS was measured on the basis of the intracellular peroxide-dependent oxidation of DCFH-DA to form the fluorescent compound 2,7-dichlorofluorescein (DCF). Cells were seeded on to a 96-well plate at a density of 30,000 cells per well and cultured overnight. Then fresh medium containing drug (5 µM, 0.04% CEL) was added and cells were incubated for 24 h in dark. After washing three times with PBS, 50 µL DCFH-DA (10 µM) was added and cells were incubated for 30 min. The old medium was discarded and washed three times with PBS followed by illumination (light dose of 1.5 J·cm^−2^) with laser light (670 nm). Then the cells were lysed with 1% SDS (100 µL) for 10 min at a table concentrator and then the DCF fluorescence was measured by a Bio-Tek microplate (Corning Incorporated., New York, NY, USA) reader (excitation/emission: 488/525 nm).

### 3.6. Cellular Uptake

The MCF-7 cells were seeded onto 96-well plates at 30,000 cells per well and incubated overnight at 37 °C under 5% CO_2_. The medium was removed and rinsed three times with PBS. Then 200 µL of the dye solution (all at 5 µM), which was prepared from DMEM culture medium with 1% DMSO and 0.04% CEL, was added. The dye solution was removed carefully after 24 h incubation and the cells were washed three times with PBS. The cells were lysed with 1% SDS (100 µL each well). Both the intracellular fluorescence caused by phthalocyanines and protein concentration, which was evaluated according to BCA protein assay kit [34] were measured by an automatic microplate reader (λ_ex_ = 610 nm, λ_em_ = 680 nm). The standard curve of each dye was obtained using cell lysates treated as above with known amounts of the appropriate phthalocyanine solution. The ability of cellular uptake is expressed as nmol phthalocyanine/mg cell protein.

### 3.7. Confocal Microscopic Analysis

Approximately 80,000 MCF-7 cells were plated on a cell culture dish (diameter = 35 mm) and incubated overnight. Then the medium was replaced by fresh medium containing drug (10 µM) and the cells were incubated for 24 h again. After incubation, the cells were rinsed with PBS three times and incubated with MitoTracker Green (Beyotime Institute of Biotechnology Co. Ltd., Haimen, China, 2 µM in culture medium, Incubated for 30 min) or LysoTracker DND-26 (Xiamen Bioluminor Bio-Technology Co. Ltd., Xiamen, China, 2 µM in culture medium, incubated for 30 min). Then the cells were rinsed with PBS three times again and examined with a scanning confocal microscope (FV1000, Olympus Instrument Co. Ltd., Shinjuku-ku, Tokyo, Japan). Specimens were excited by laser at 488 nm (Mito-Tracker Green, Lyso-Tracker Green DND 26), or 633 nm (phthalocyanines), and the fluorescence was collected for the formers (green, 510−570 nm), and the latters (red, 650–750 nm). The subcellular localization of glucosylated phthalocyanines **7a**–**7b** and the references **6a**–**6b** was revealed by comparing the intracellular fluorescence images caused by the fluorescent probe and phthalocyanines. The competitive uptake test was carried out according to the procedure described above. The difference is that MCF-7 (80,000) and HELF (120,000) cells were grown by means of co-culture.

## 4. Conclusions

We have successfully synthesized and characterized two novel mono-glucosylated zinc(II) phthalocyanines **7a**–**7b**, as well as the acetyl-protected counterparts **6a**–**6b**, via the Cu(I)-catalyzed click reaction. Their photo-physical, photo-chemical and photo-biological properties have also been explored. The results show that all of them are highly soluble and slightly aggregated in the biological media; phthalocyanines **7a** and **7b** bearing glucose moiety have a specific affinity to MCF-7 breast cancer cells over HELF normal cells; furthermore, both compounds are localized in the lysosome and exhibit high photocytotoxicity towards MCF-7 cells with the IC_50_ value as low as 0.32 µM; all of these results show that the glucosylated zinc(II) phthalocyanines **7a**–**7b** are highly promising antitumor agents for targeting photodynamic therapy.

## Figures and Tables

**Table 1:** Photo-physical/photo-chemical data of glucosylated zinc(II) phthalocyanines in DMF.

Compounds	λ_max_/nm (log ε)	λ_em_/nm ^a^	Φ_F_^b^	Φ_Δ_^c^
**6a**	672 (5.29)	680	0.26	0.65
**6b**	672 (5.29)	681	0.26	0.65
**7a**	672 (5.28)	680	0.26	0.63
**7b**	672 (5.31)	681	0.28	0.65

^a^ Excited at 610 nm. ^b,c^ Using ZnPc in DMF as the reference (Φ_F_ = 0.28, Φ_Δ_ = 0.56).

**Table 2:** IC_50_ values of **6a**–**6b** and **7a**–**7b** against MCF-7 cells with the light dose of 1.5 J/cm^2^.

Compounds	6a	6b	7a	7b
**IC_50_**	0.010	0.011	0.032	0.041

## Supplementary Materials

Supplementary materials are available online.

## References

[1] Dolmans D.E.J.G.J., Fukumura D., Jain R.K. (2003). Photodynamic therapy for cancer. Nat. Rev. Cancer.

[2] Castano A.P., Mroz P., Hamblin M.R. (2006). Photodynamic therapy and anti-tumour immunity. Nat. Rev. Cancer.

[3] Celli J.P., Spring B.Q., Rizvi I., Evans C.L., Samkoe K.S., Verma S., Pogue B.W., Hasan T. (2010). Imaging and Photodynamic Therapy: Mechanisms, Monitoring, and Optimization. Chem. Rev..

[4] Ali H., van Lier J.E. (1999). Metal complexes as photo-and radiosensitizers. Chem. Rev..

[5] Lukyanets E.A. (1999). Phthalocyanines as photosensitizers in the photodynamic therapy of cancer. J. Porphyr. Phthalocyanines.

[6] Allen C.M., Sharman W.M., Van Lier J.E. (2001). Current status of phthalocyanines in the photodynamic therapy of cancer. J. Porphyr. Phthalocyanines.

[7] Ogura S., Tabata K., Fukushima K., Kamachi T., Okura I. (2006). Development of phthalocyanines for photodynamic therapy. J. Porphyr. Phthalocyanines.

[8] Taquet J.P., Frochot C., Manneville V., Barberi-Heyob M. (2007). Phthalocyanines covalently bound to biomolecules for a targeted photodynamic therapy. Curr. Med. Chem..

[9] Zhang C., Gao L., Cai Y., Liu H., Gao D., Lai J., Jia B., Wang F., Liu Z. (2016). Inhibition of tumor growth and metastasis by photoimmunotherapy targeting tumor-associated macrophage in a sorafenib-resistant tumor model. Biomaterials.

[10] Chen Z., Xu P., Chen J., Chen H., Hu P., Chen X., Lin L., Huang Y., Zheng K., Zhou S. (2014). Zinc phthalocyanine conjugated with the amino-terminal fragment of urokinase for tumor-targeting photodynamic therapy. Acta Biomater..

[11] Kamarulzaman E.E., Gazzali A.M., Acherar S., Frochot C., Barberi-Heyob M., Boura C., Chaimbault P., Sibille E., Wahab H.A., Vanderesse R. (2015). New peptide-Conjugated Chlorin-Type Photosensitizer Targeting Neuropilin-1 for Anti-Vascular Targeted Photodynamic Therapy. Int. J. Mol. Sci..

[12] Li S.-Y., Cheng H., Qiu W.-X., Liu L.-H., Chen S., Hu Y., Xie B.-R., Li B., Zhang X.-Z. (2015). Protease-Activable Cell-Penetrating Peptide-Protoporphyrin Conjugate for Targeted Photodynamic Therapy in Vivo. ACS Appl. Mater. Interfaces.

[13] Mallikaratchy P., Tang Z.W., Tan W.H. (2008). Cell specific aptamer-photosensitizer conjugates as a molecular tool in photodynamic therapy. ChemMedChem.

[14] Gao Y., Qiao G.M., Zhuo L.H., Li N., Liu Y., Tang B. (2011). A tumor mRNA-mediated bi-photosensitizer molecular beacon as an efficient imaging and photosensitizing agent. Chem. Commun..

[15] Schneider R.L., Schmitt F., Frochot C., Fort Y., Lourette N., Guillemin F., Muller J.F., Barberi-Heyob M. (2005). Design, synthesis, and biological evaluation of folic acid targeted tetraphenylporphyrin as novel photosensitizers for selective photodynamic therapy. Bioorg. Med. Chem..

[16] Ke M.R., Yeung S.L., Ng D.K.P., Fong W.P., Lo P.C. (2013). Preparation and in Vitro Photodynamic Activities of Folate-Conjugated Distyryl Boron Dipyrromethene Based Photosensitizers. J. Med. Chem..

[17] Stallivieri A., Colombeau L., Jetpisbayeva G., Moussaron A., Myrzakhmetov B., Arnoux P., Acherar S., Vanderesse R., Frochot C. (2017). Folic acid conjugates with photosensitizers for cancer targeting in photodynamic therapy: Synthesis and photophysical properties. Bioorg. Med. Chem..

[18] Goksel M. (2016). Synthesis of asymmetric zinc(II) phthalocyanines with two different functional groups & spectroscopic properties and photodynamic activity for photodynamic therapy. Bioorg. Med. Chem..

[19] Shin W.S., Han J., Kumar R., Lee G.G., Sessler J.L., Kim J.H., Kim J.S. (2016). Programmed activation of cancer cell apoptosis: A tumor-targeted phototherapeutic topoisomerase I inhibitor. Sci. Rep..

[20] Zhang F.L., Huang Q., Zheng K., Li J., Liu J.Y., Xue J.P. (2013). A novel strategy for targeting photodynamic therapy. Molecular combo of photodynamic agent zinc(II) phthalocyanine and small molecule target-based anticancer drug erlotinib. Chem. Commun..

[21] Airley R.E., Mobasheri A. (2007). Hypoxic regulation of glucose transport, anaerobic metabolism and angiogenesis in cancer: Novel pathways and targets for anticancer therapeutics. Chemotherapy.

[22] Singh S., Aggarwal A., Bhupathiraju N.V.S.D.K., Arianna G., Tiwari K., Drain C.M. (2015). Glycosylated Porphyrins, Phthalocyanines, and Other Porphyrinoids for Diagnostics and Therapeutics. Chem. Rev..

[23] Bachle F., Hanack M., Ziegler T. (2015). Synthesis and Spectroscopic Evaluation of Two Novel Glycosylated Zinc(II)-Phthalocyanines. Molecules.

[24] Mou T.T., Zhao Z.Q., Zhang P., Fang W., Peng C., Lu J., Wang Q., Ma Y.C., Zhang X.Z. (2015). Synthesis and Bio-Evaluation of New F-18-Labeled Pyridaben Analogs with Improved Stability for Myocardial Perfusion Imaging in Mice. Chem. Biol. Drug. Des..

[25] Brunner K., Harder J., Halbach T., Willibald J., Spada F., Gnerlich F., Sparrer K., Beil A., Mockl L., Brauchle C. (2015). Cell-Penetrating and Neurotargeting Dendritic siRNA Nanostructures. Angew. Chem. Int. Ed..

[26] Scalise N., Durantini E.N. (2005). Synthesis, properties, and photodynamic inactivation of Escherichia coli using a cationic and a noncharged Zn(II) pyridyloxyphthalocyanine derivatives. Bioorg. Med. Chem..

[27] Maree M.D., Kuznetsova N., Nyokong T. (2001). Silicon octaphenoxyphthalocyanines: Photostability and singlet oxygen quantum yields. J. Photochem. Photobiol. A.

[28] Ogunsipe A., Nyokong T. (2004). Effects of substituents and solvents on the photochemical properties of zinc phthalocyanine complexes and their protonated derivatives. J. Mol. Struct..

[29] Robertson C.A., Evans D.H., Abraharnse H. (2009). Photodynamic therapy (PDT): A short review on cellular mechanisms and cancer research applications for PDT. J. Photochem. Photobiol. B.

[30] Wang J., Yi J. (2008). Cancer cell killing via ROS To increase or decrease, that is the question. Cancer Biol. Ther..

[31] Chen X.P., Zhong Z.F., Xu Z.T., Chen L.D., Wang Y.T. (2010). 2′,7′-Dichlorodihydrofluorescein as a fluorescent probe for reactive oxygen species measurement: Forty years of application and controversy. Free Radic. Res..

[32] Salman A.A., Heidelberg T. (2014). In situ functionalized fluorescent nanoparticles for efficient receptor coupling. J. Nanopart. Res..

[33] Gerlier D., Thomasset N. (1986). Use of Mtt Colorimetric Assay to Measure Cell Activation. J. Immunol. Methods.

[34] Hofstraat J.W., Latuhihin M.J. (1994). Correction Of Fluorescence-Spectra. Appl. Spectrosc..

