# Supplementary material for: Preparation and In Vitro Photodynamic Activity of Glucosylated Zinc(II) Phthalocyanines as Underlying Targeting Photosensitizers
Source: Molecules. 2017 May 19;22(5):845. doi: 10.3390/molecules22050845 (PMC6154674; doi:10.3390/molecules22050845)
Supplement: Supplementary file 1 [file molecules-22-00845-s001.pdf]

Electronic Supporting Information

Preparation and in Vitro Photodynamic Activity of Glucosylated Zinc(Ⅱ) Phthalocyanines as Underlying Targeting Photosensitizers

Jian-Yong Liu [1,*], Chen Wang [1], Chun-Hui Zhu [1], Zhi-Hong Zhang [2,*] and Jin-Ping Xue[1]

[1] State Key Laboratory of Photocatalysis on Energy and Environment & National & Local Joint Biomedical Engineering Research Center on Photodynamic Technologies, College of Chemistry, Fuzhou University, Fuzhou 350108, P. R. China
[2] Fuzhou General Hospital of Nanjing Military Command, Fuzhou 350005, P.R. China

Table of Contents

Figure S1. ^{1}H NMR spectrum of **2**

Figure S2. ^{13}C NMR spectrum of **2**

Figure S3. HRMS spectrum of **2**

Figure S4. ^{1}H NMR spectrum of **3**

Figure S5. HRMS spectrum of **3**

Figure S6. ^{1}H NMR spectrum of **6a**

Figure S7. HRMS spectrum of **6a**

Figure S8. ^{1}H NMR spectrum of **6b**

Figure S9. HRMS spectrum of **6b**

Figure S10 ^{1}H NMR spectrum of **7a**

Figure S11 HRMS spectrum of **7a**

Figure S12 ^{1}H NMR spectrum of **7b**

Figure S13 HRMS spectrum of **7b**

Residual solvent signals are marked with asterisks in all of the following spectra.

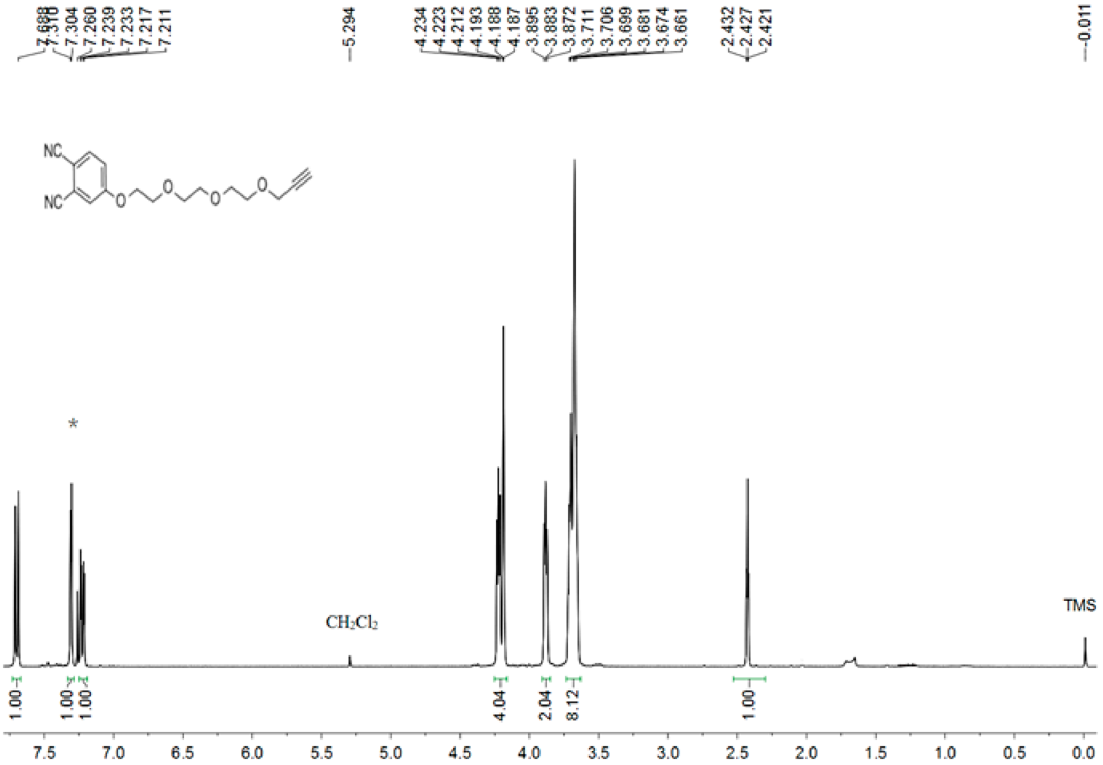

Figure S1. ^{1}H NMR spectrum of compound **2** in $CDCl_3$.

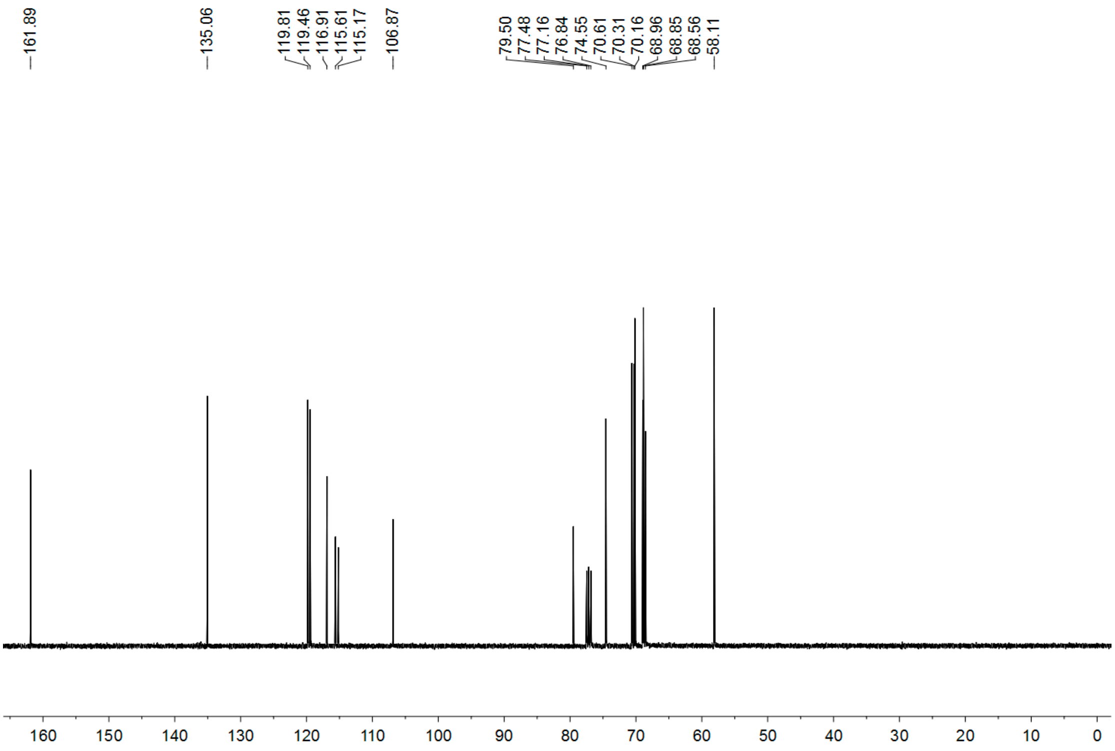

Figure S2. ^{13}C NMR spectrum of compound **2** in $CDCl_3$.

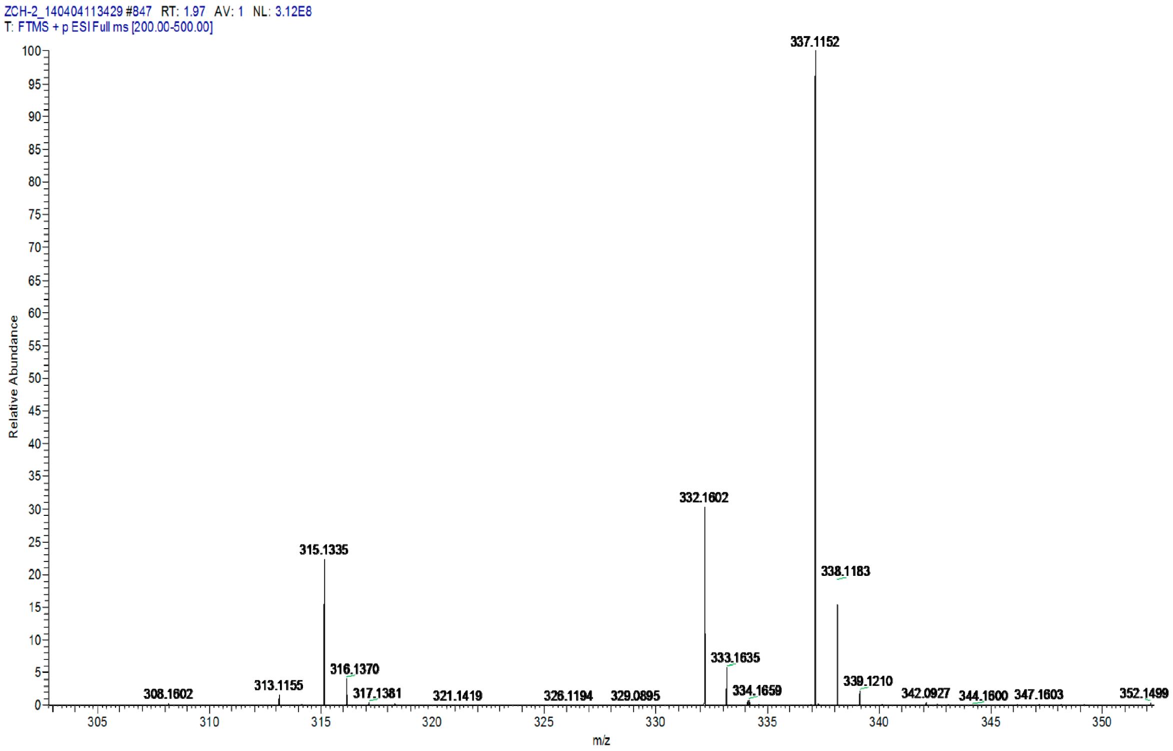

Figure S3. HRMS spectrum of compound **2**.

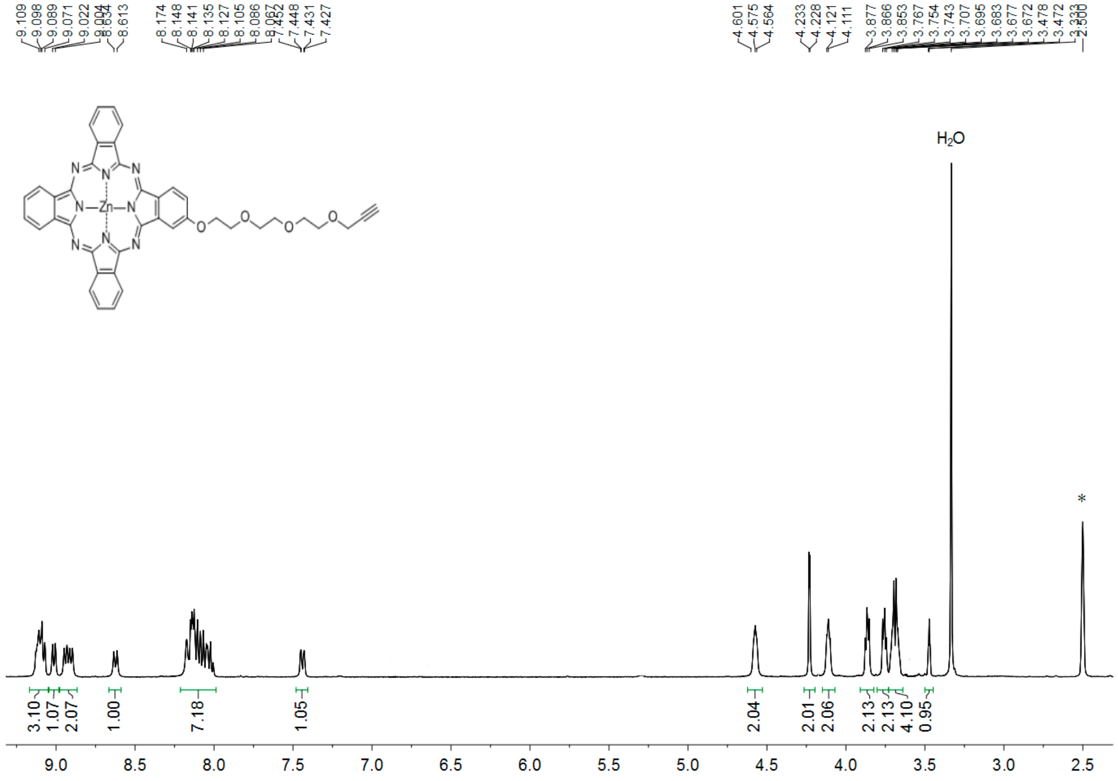

Figure S4. ^{1}H NMR of compound **3** in DMSO-d$_6$.

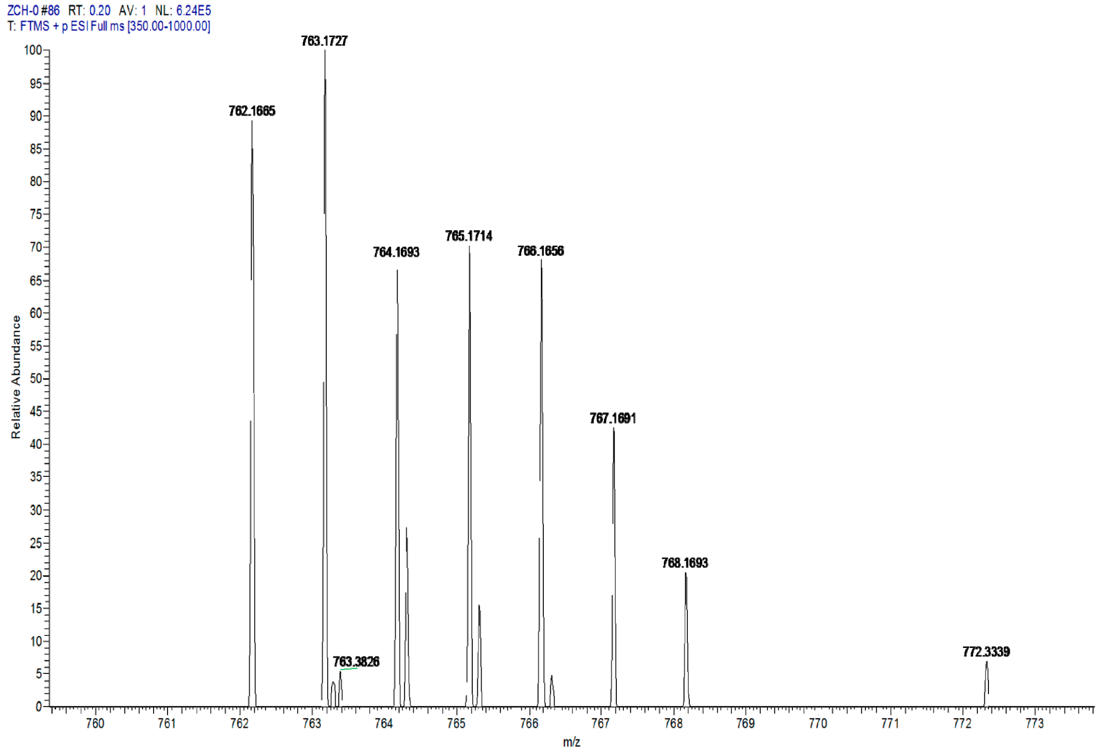

Figure S5. HRMS spectrum of compound **3**.

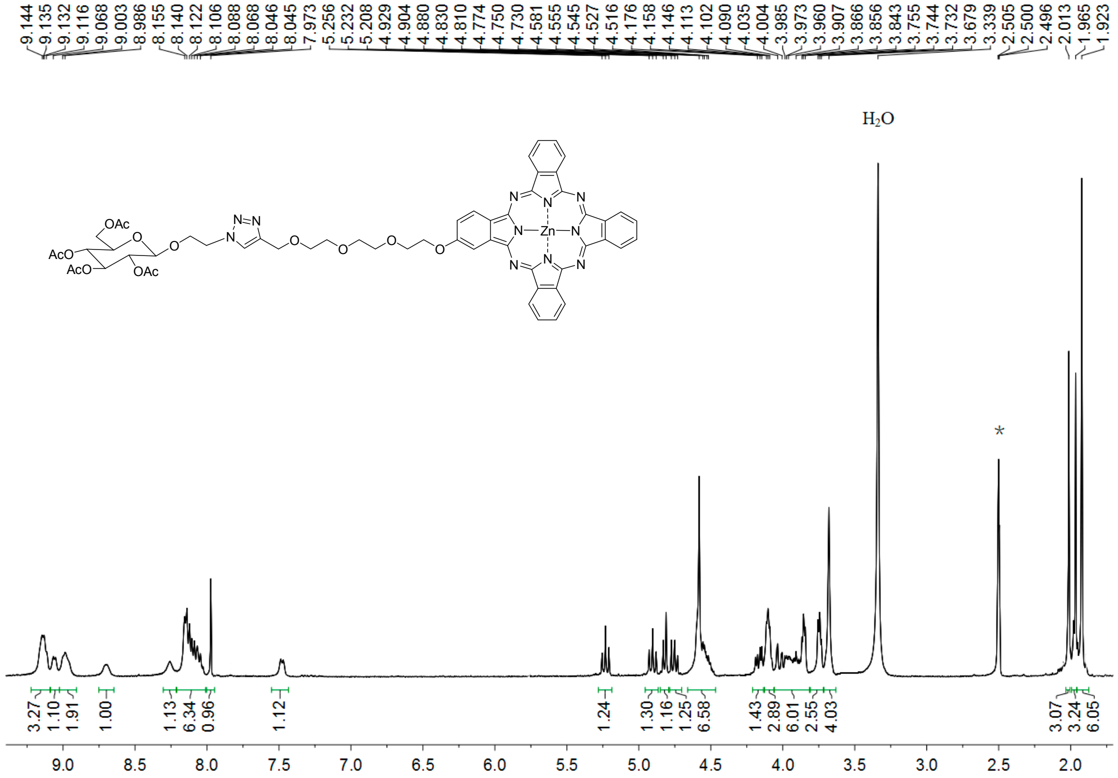

Figure S6. ^{1}H NMR spectrum of compound **6a** in DMSO-d_6.

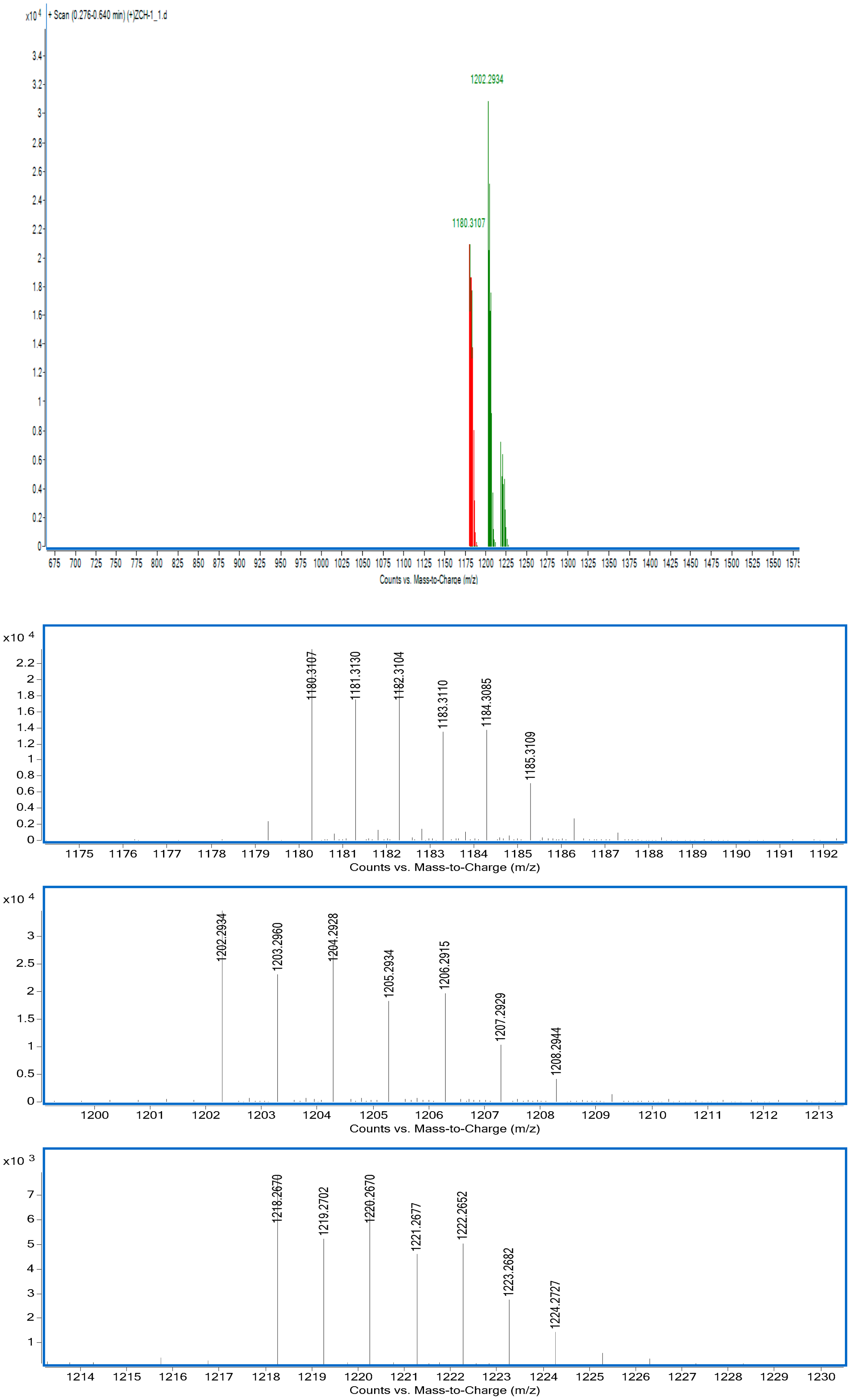

Figure S7. HRMS spectrum of compound **6a**.

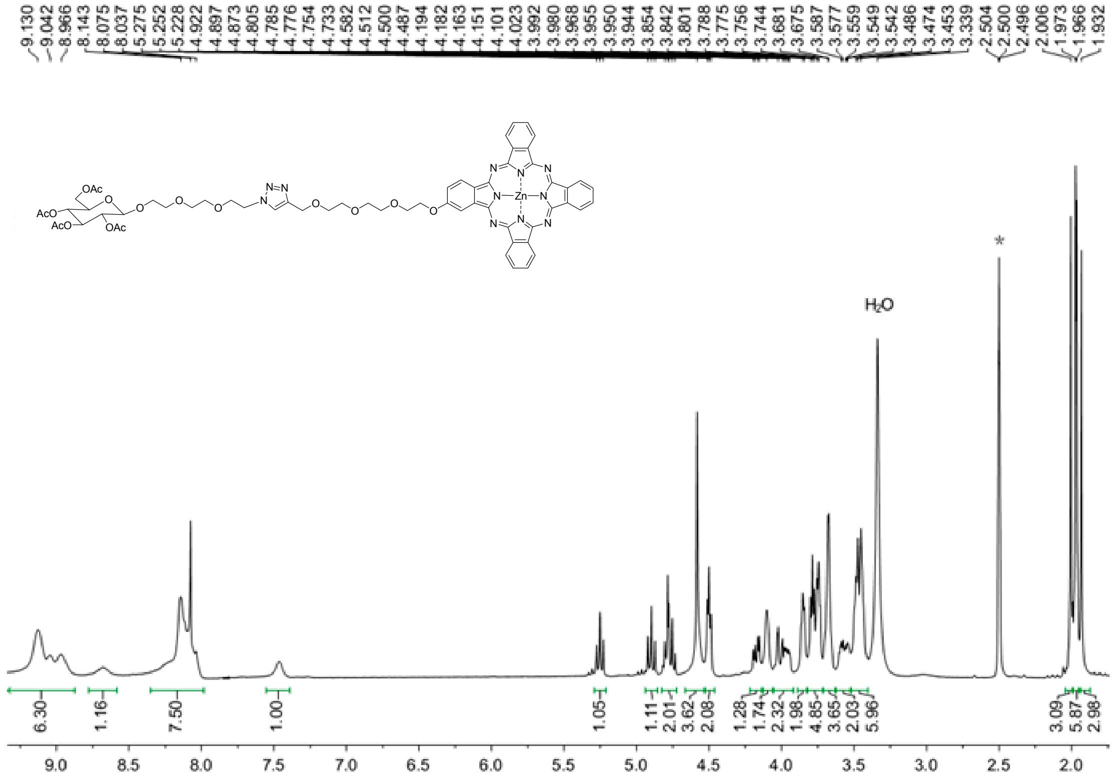

Figure S8. ^{1}H NMR spectrum of compound **6b** in DMSO-d_6.

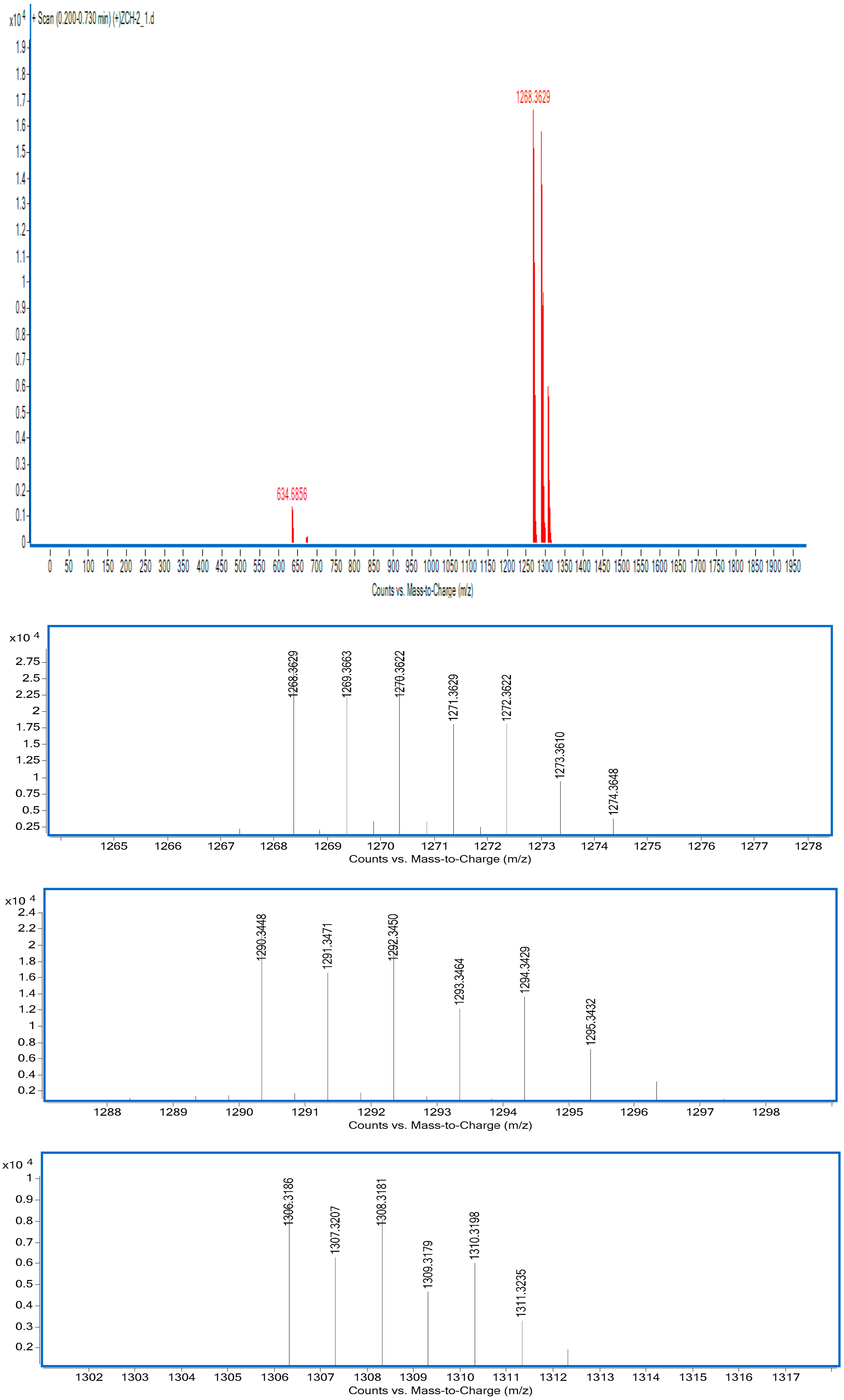

Figure S9. HRMS spectrum of compound **6b**.

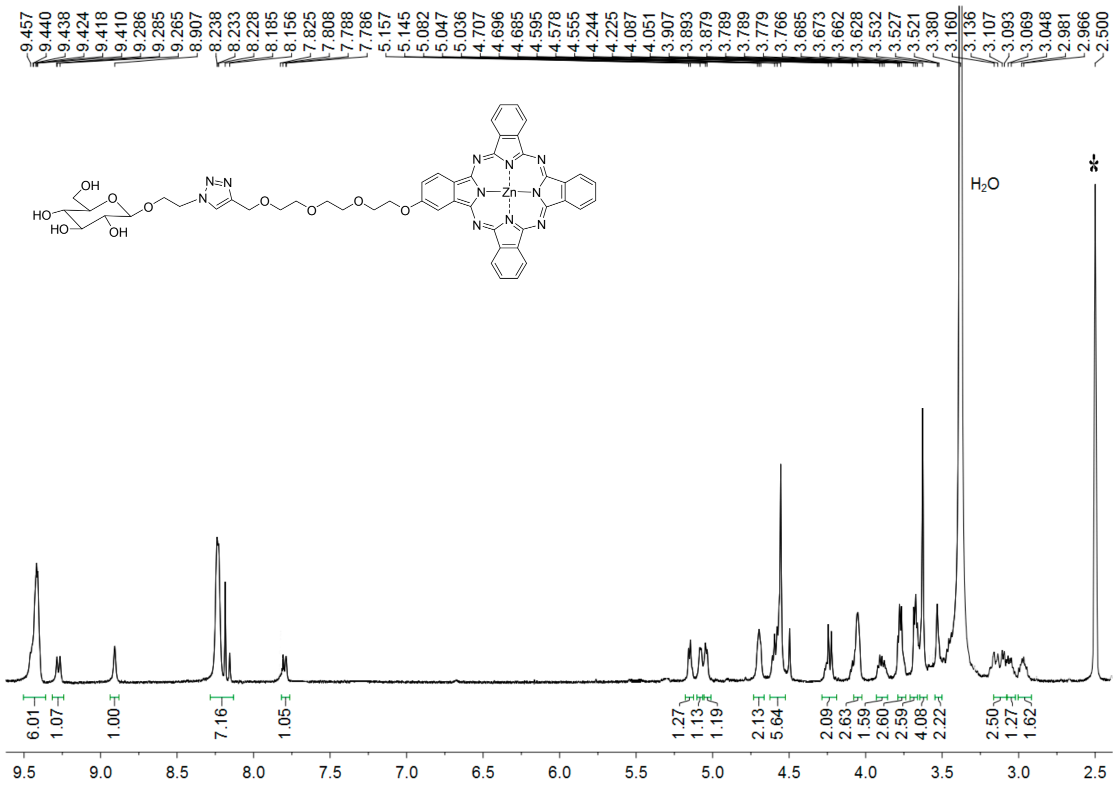

Figure S10. ^1H NMR spectrum of compound **7a** in DMSO-d$_6$.

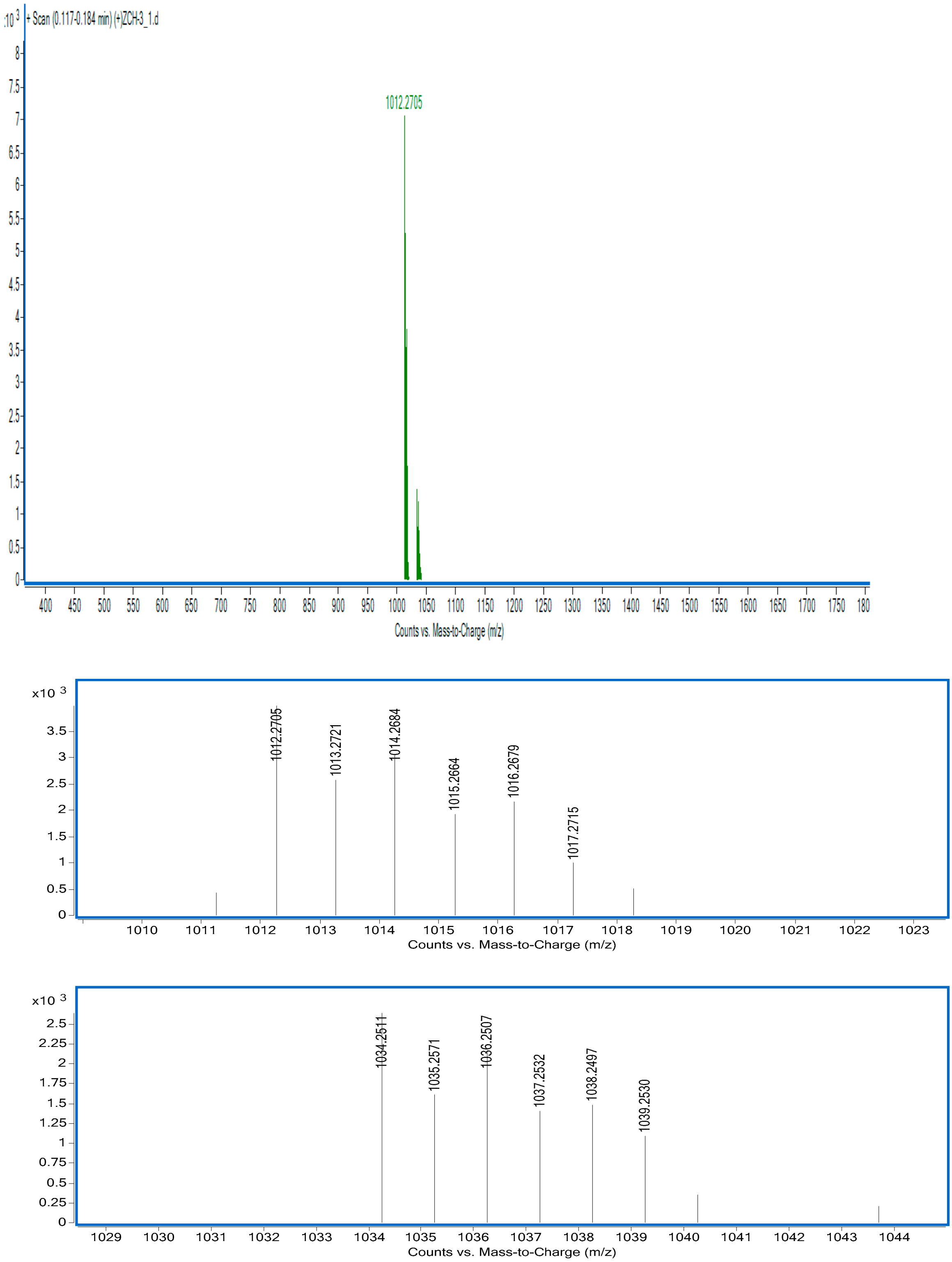

Figure S11. HRMS spectrum of compound **7a**.

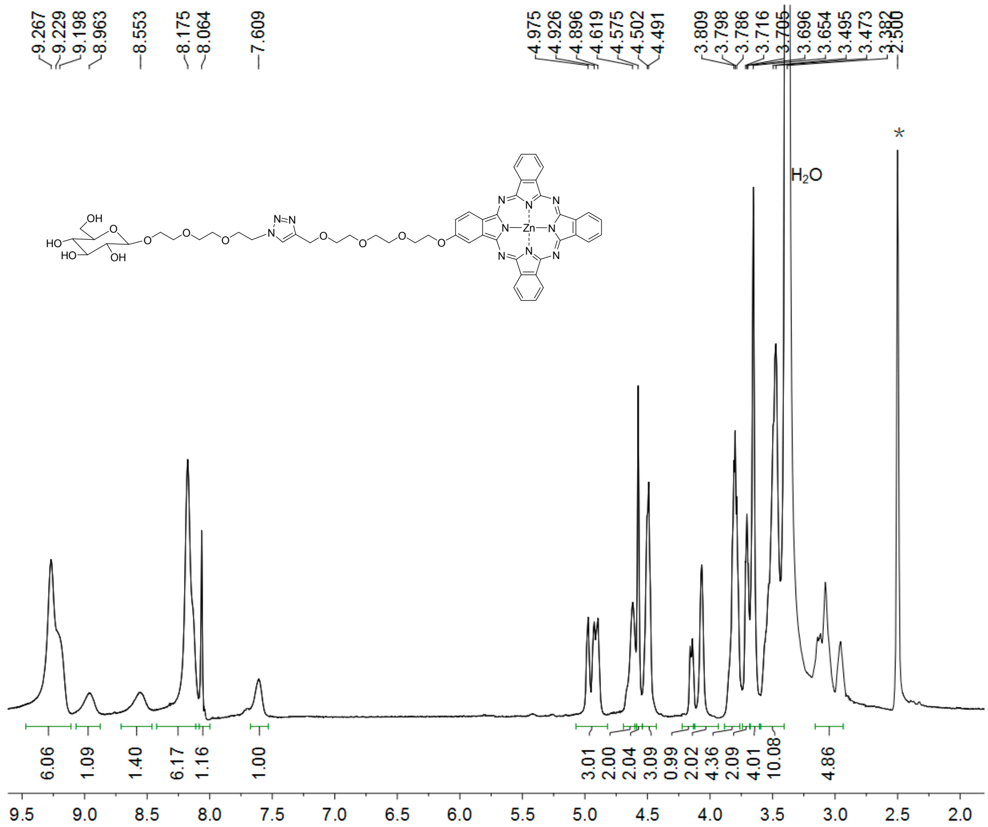

Figure S12. ^{1}H NMR spectrum of compound **7b** in DMSO-d_6.

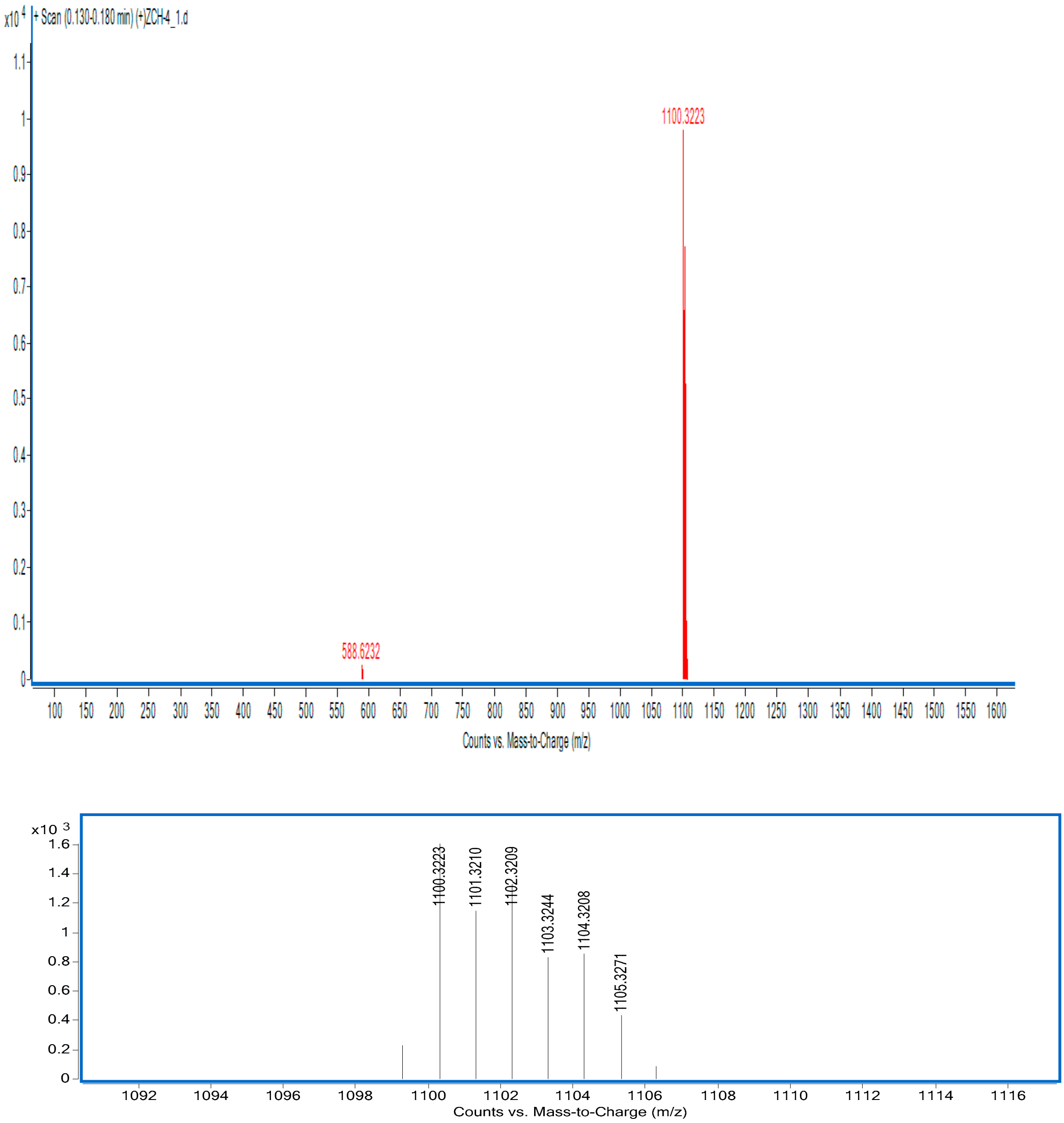

Figure S13. HRMS spectrum of compound **7b**.
